# Development and Validation of a Prognostic Signature Based on Immune Genes in Cervical Cancer

**DOI:** 10.3389/fonc.2021.616530

**Published:** 2021-03-17

**Authors:** Yu Chen, Hao Lin, Ya-Nan Pi, Xi-Xi Chen, Hu Zhou, Yuan Tian, Wei-Dong Zhao, Bai-Rong Xia

**Affiliations:** ^1^ Department of Obstetrics and Gynecology, The First Affiliated Hospital of USTC, Division of Life, Sciences and Medicine, University of Science and Technology of China, Hefei, China; ^2^ Graduate School, Benbu Medical College, Benbu, China; ^3^ Department of Gynecology, Harbin Medical University Cancer Hospital, Harbin, China

**Keywords:** cervical cancer, immune signature, immune-based prognostic score, immune genes, prognostic signature

## Abstract

**Background:**

Cervical cancer is one of the most common types of gynecological malignancies worldwide. This study aims to develop an immune signature to predict survival in cervical cancer.

**Method:**

The gene expression data of 296 patients with cervical cancer from The Cancer Genome Atlas database (TCGA) and immune-related genes from the Immunology Database and Analysis Portal (*ImmPort*) database were included in this study. The immune signature was developed based on prognostic genes. The validation dataset was downloaded from the Gene Expression Omnibus (GEO) database.

**Result:**

The immune signature namely immune-based prognostic score (IPRS) was developed with 229 genes. Multivariate analysis revealed that the IPRS was an independent prognostic factor for overall survival (OS) and progression-free survival (PFS) in patients with cervical cancer. Patients were stratified into high IPRS and low IPRS groups, and those in the high IPRS group were associated with better survival, which was validated in the validation set. A nomogram with IPRS and stage was constructed to predict mortality in cervical cancer.

**Conclusions:**

We developed a robust prognostic signature IPRS that could be used to predict patients’ survival outcome.

## Introduction

With an estimated 570,000 new cervical cancer cases and 311,000 deaths worldwide in 2018, cervical cancer is one of the most common types of gynecological malignancies and ranks as the fourth most frequently diagnosed cancer and the fourth leading cause of cancer-related death in women (1). There are two primary histological types of cervical cancer: cervical squamous cell carcinoma and cervical adenocarcinoma. In less developed countries, cervical cancer among women is the leading cause of cancer death, and nearly 90% of cervical cancer deaths occurred in developing countries (1). Although the incidence is gradually decreasing owing to the identification of HPV as an etiologic factor and the introduction of a specific vaccine, the prognosis of advanced stage disease is extremely poor (2). Various biomarkers, especially genetic markers, have been shown to be closely related to prognosis (3–7). Thus, identifying patients with poor prognosis and high mortality is an important basis for additional clinical therapy.

The immune system has been found to be a determining factor during cancer initiation and progression and immunotherapy has shown great promise for some cancers. Meanwhile, evidence has shown that immunotherapy plays an important role in cervical cancer because the immune reaction to HPV may inhibit further progression in early-stage cancer (2). Further evidence has preliminarily confirmed that several immune prognostic signatures could be used to predict the prognosis of cervical cancer (8, 9). Thus, immune prognostic signatures have therapeutic potential in cervical cancer.

In this study, we aimed to develop a new immune signature with immune prognostic genes. The new immune signature was developed in a training set and validated in a testing set. In an independent cohort, we proved the signature stability and reliability. Further, we used subgroup analysis and sensitivity analysis to validate the new immune signature. Additionally, with the new immune signature and clinical characteristics, we built a nomogram for patient prediction for clinical research.

## Materials and Methods

### Study Population and Eligibility Criteria

**Figure 1:**
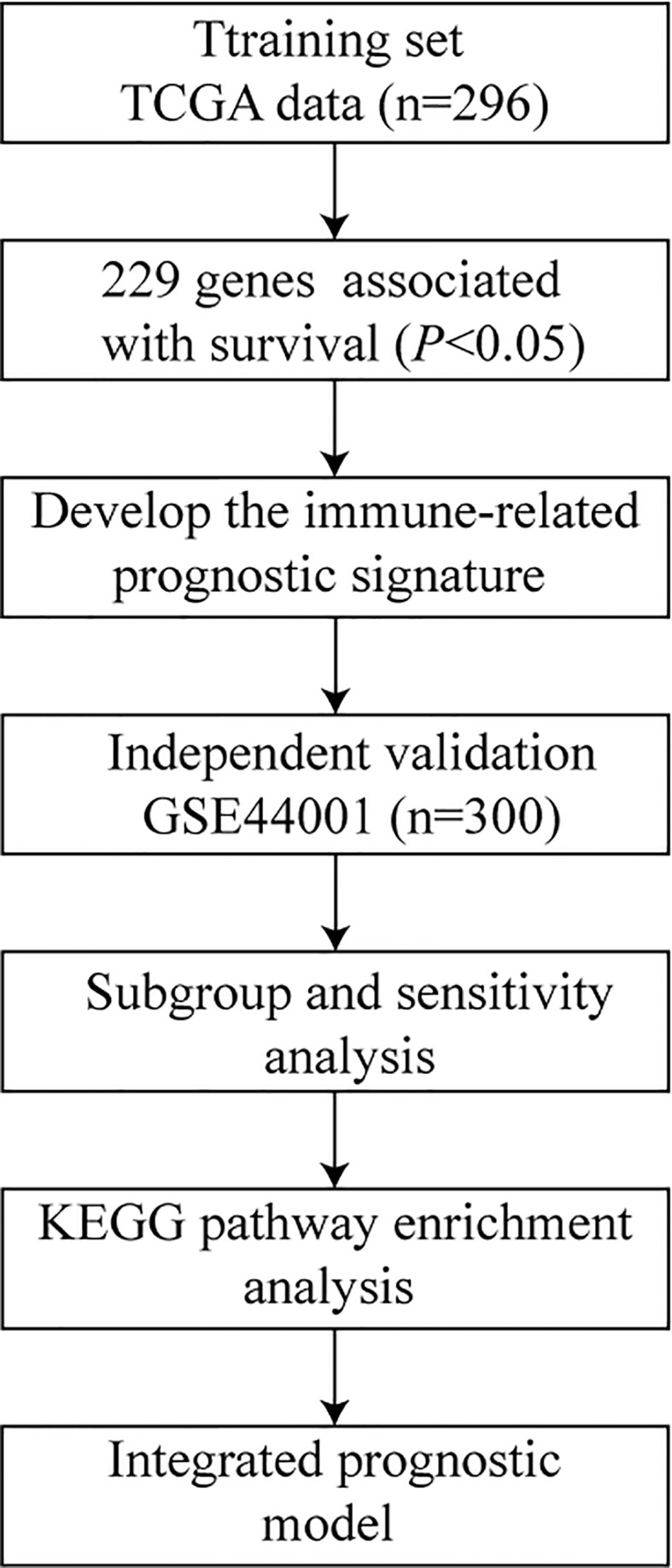
Flowchart of the study.

We collected the cervical cancer gene expression profiles of primary tumor tissue samples from public datasets, including a cohort GSE44001 from the Gene Expression Omnibus (GEO) (https://www.ncbi.nlm.nih.gov/geo/) and another from The Cancer Genome Atlas (TCGA) (https://portal.gdc.cancer.gov/). Only patients with follow-up duration and status were included. In this study, the outcomes were overall survival (OS) and progression-free survival (PFS). Finally, 596 cervical cancer cases including 296 from TCGA and 300 from GEO were included. The study design is presented in **Figure 1**.

### Gene Expression Data Preprocessing

For the GEO dataset, gene microarray data and clinical information were downloaded from GEO. The missing gene expression was filled by k-nearest neighbors with R package “*impute*” (10). For the TCGA dataset, RNA sequencing data (FPKM value) of gene expression and clinical information were also downloaded. Then, the gene expression of each gene in the two datasets was transformed into a *z-score*.

### Immune-Related Genes Definition

The comprehensive list of immune-related genes was download from the Immunology Database and Analysis Portal (*ImmPort*) database (https://immport.niaid.nih.gov), which includes 17 immune categories about molecular function (11). By matching with the variables from GSE44001 and TCGA, a total of 1267 immune-related genes were included in this study.

### Development of the Immune-Based Prognostic Signature for Cervical Cancer

We developed the signature called immune-based prognostic score (IPRS) through a two-stage strategy. First, the univariable Cox proportional hazards regression analysis was used to assess the association of 1267 immune genes with OS in TCGA. Those genes with significant prognosis were extracted for further analysis. Then, we defined the immune-based prognostic signature similar to GGI (12):

$$IPRS=\sum_{i\in a}gene_{i}-\sum_{j\in b}gene_{j},$$

where *i* is the risky gene whose Cox coefficient is positive, and *j* is the protective gene whose Cox coefficient is negative.

### Validation of the IPRS

To obtain a uniform cut-off value to divide patients into high-score and low-score groups, the cut-off of IPRS was determined by using the “ surv-cutpoint” function of the R package “survmier,” which repeatedly tested all potential cut-off points to determine the maximum rank statistic (13). Then, the IPRS was further validated in GSE44001.

### Functional Enrichment Analyses

To further investigate the gene molecular mechanisms in IRRS, Kyoto Encyclopedia of Genes and Genomes (KEGG) enrichment analyses was performed by using R package “clusterProfiler”. The *P* values adjusted by False-Discovery Rate (FDR) <0.05 were considered significant.

### Construction and Validation of a Predictive Nomogram

We used a multivariate cox proportional hazard model to determine independent prognostic factors that were used to establish a nomogram with the R package “rms” (14). The calibration curves were used to determine whether the nomogram was suitable for clinical use.

### Statistical Analysis

Categorical measurements were described as count and percentage, while continuous measurements were presented as mean ± SD. Kaplan–Meier (KM) survival curves were drawn using “survmier” and compared between subgroups using the log-rank test. Multivariate cox proportional hazard models were used to estimate the hazard ratios of variables and determine independent prognostic factors. The C-index was estimated using “survival” in the R package. All statistical analyses were performed in R version 3.3.4 (http://www.r-project.org/). A two-sided P<0.05 was considered to indicate statistical significance.

## Results

### Development and Definition of the IPRS

According to the inclusion criteria, a total of 596 cervical cancer patients including 296 patients from TCGA and 300 patients from GSE44001 were included in this study (**Table 1**). In the training set, 143 protective genes and 86 risky genes among the 1267 immune-related genes were associated with OS (**Table S1**).

**Table 1:** Clinical characteristics of cervical cancer patients.

	TCGA	GSE44001
Number of samples	296	300
Survival event		–
Alive	229 (77.4)	–
Dead	61 (22.6)	–
PFS event^*^		
Did not occur	232 (78.4)	262(87.3)
Occurred	64 (21.6)	38(12.7)
Age	48.33 ± 13.83	–
Stage		
I	157 (53.0)	258 (86.0)
II	68 (23.0)	42 (14.0)
III	43 (14.5)	0
IV	21 (7.1)	0
Unknown	7 (2.4)	0
Grade		–
I/II	146 (49.3)	–
III/IV	118 (39.9)	–
Unknown	32 (10.8)	–

^*^The PFS time of 18 patients is missing.

Then, we used these genes to develop IPRS whose cut-off was -74.30 to stratify patients into high IPRS and low IPRS groups in the study.

### Validation of the IPRS

**Figure 2:**
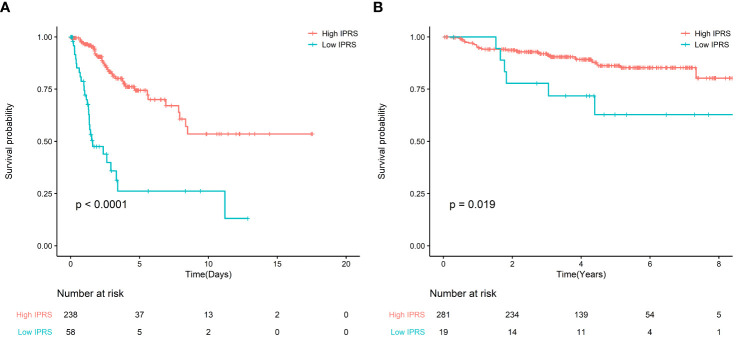
**(A)** Survival curve of OS between high IRPS and low IRPS in TCGA; **(B)** Survival curve of FPS between high IRPS and low IRPS in GSE44001.

For the TCGA cohort, the KM curves indicated that the high IPRS group was associated with better OS, while the low IPRS group was associated with poor OS (**Figure 2A**). After adjusting for age, stage, and grade, IPRS (HR: 4.07, 95% CI: 2.29–7.23) remained an independent prognostic factor in the multivariable Cox model (**Table S2**). In addition, for GSE44001, adjusting for stage and low IPRS showed a 2.67-fold (HR: 2.67, 95% CI: 1.11–6.39) higher risk than the high IPRS group (**Figure 2B**, **Table S3**).

### Subgroups and Sensitivity Analysis for IPRS

**Figure 3:**
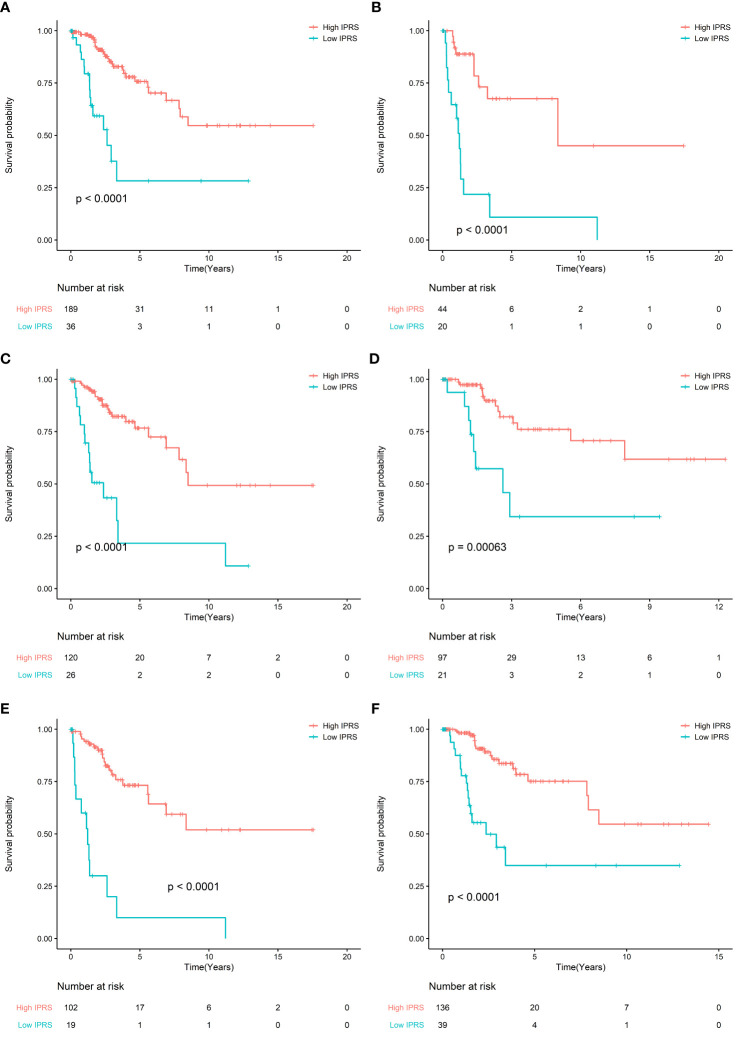
**(A)** Survival curve of OS between high IRPS and low IRPS in stage I and stage II patients; **(B)** Survival curve of OS between high IRPS and low IRPS in stage III and stage IV patients. **(C)** Survival curve of OS between high IRPS and low IRPS in Grade I and Grade II patients; **(D)** Survival curve of OS between high IRPS and low IRPS in Grade III and Grade IV patients. **(E)** Survival curve of OS between high IRPS and low IRPS in patients aged ≤50 years; **(F)** Survival curve of OS between high IRPS and low IRPS in patients aged ≤50 years.

To evaluate the prognostic value of IPRS, we performed a sensitivity analysis according to age, stage, grade. We found that IPRS was still significant in all subgroups and the prognosis of high IPRS is better than low IPRS. These results indicated that IPRS was potentially prognostic factor (**Figure 3**). In addition, the survival curves of PFS in the TCGA cohort also showed that high IPRS was associated with better prognosis, although some information of time and status related to PFS is missing (**Figure S1**, **Table S4**).

### Pathway Enrichment Analysis

**Figure 4:**
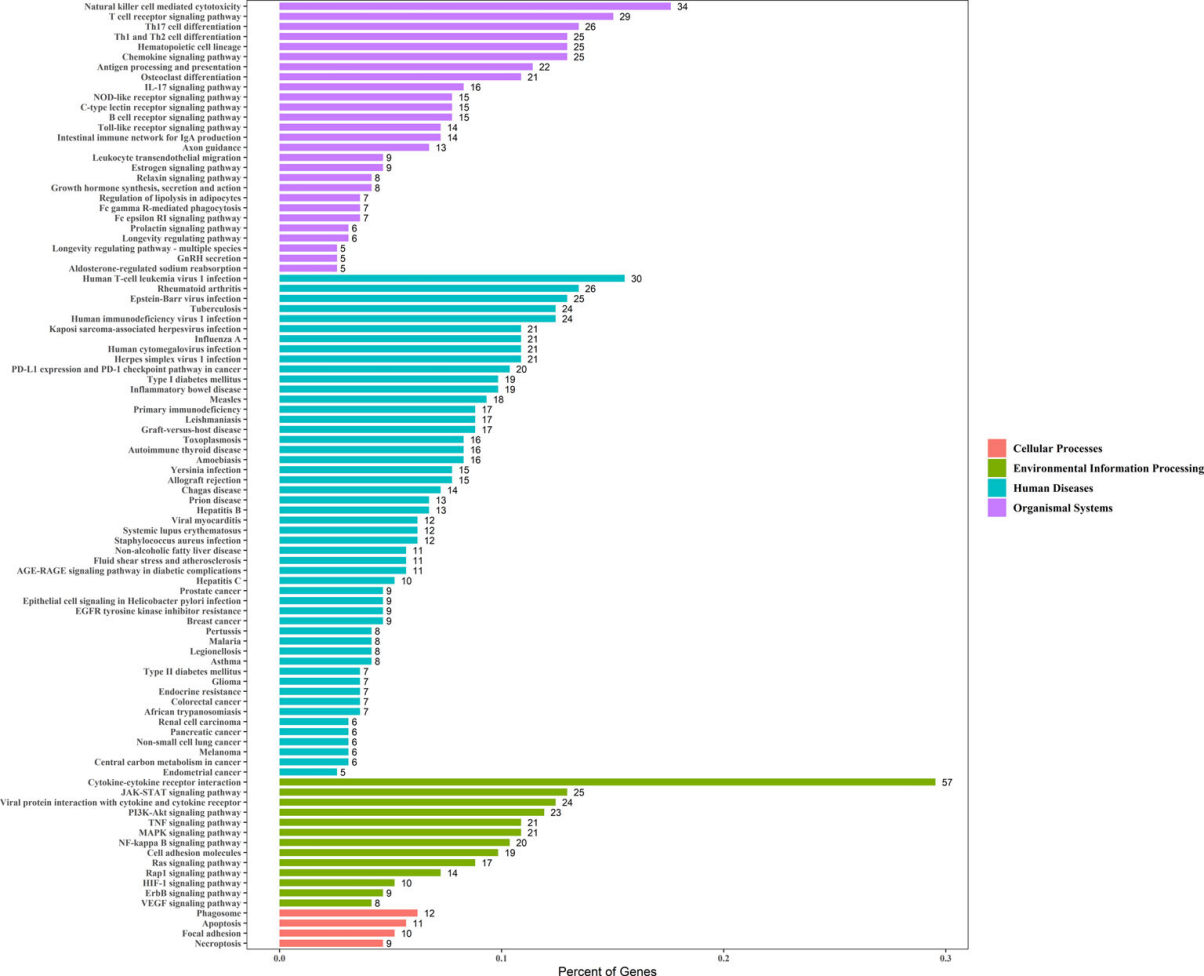
The X-axis represents the Percent of Genes. The Y-axis represents the KEGG pathway terms and the KEGG pathway terms were assigned to four KEGG categories.

Enrichment analysis of the 229 genes identified 94 significant KEGG pathways, and the top six most significant enriched pathways by these prognostic genes were: cytokine-cytokine receptor interaction, natural killer cell mediated cytotoxicity, T cell receptor signaling pathway, rheumatoid arthritis, type I diabetes mellitus, and Th1 and Th2 cell differentiation (**Figure 4**).

### Comparison With Clinical Characteristics and Constructing Nomogram

**Figure 5:**
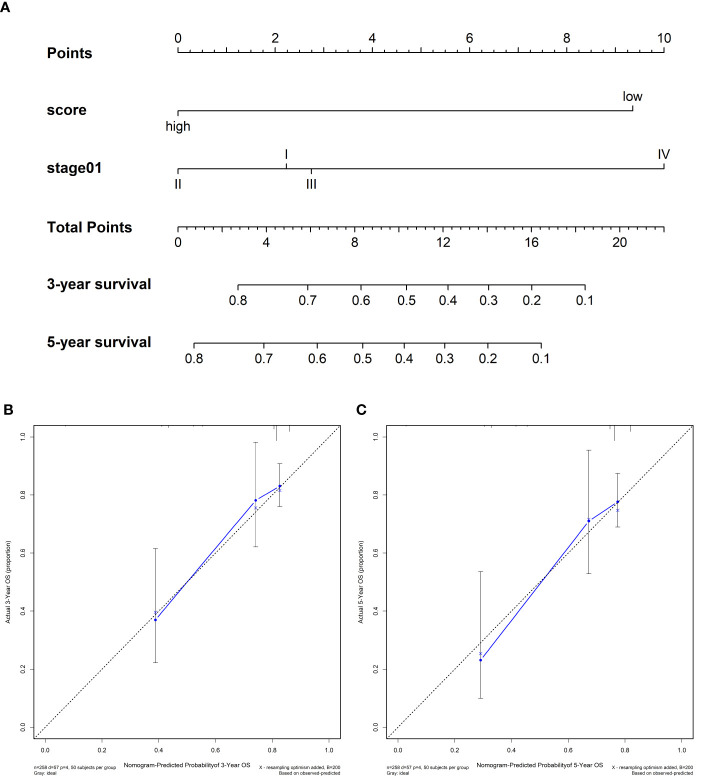
**(A)** Nomograms for predicting the probability of patient mortality at 3- or 5-year OS based on IPRS. **(B, C)** Calibration curves of the nomogram for predicting the probability of OS at 3- and 5-years.

We compared the predictive accuracy of IPRS with the clinicopathological characteristics including age, stage, and grade in TCGA. We found that compared with age (0.556), stage (0.629), and grade (0.514), IPRS had the highest C-index (0.685). In addition, stage was also a prognostic factor for predicting cervical cancer survival. We constructed a nomogram with IPRS and stage by which clinicians predict mortality in cervical cancer patients (**Figure 5A**). Furthermore, calibration curves indicated good predictive performance of the nomogram (**Figure 5B, C**).

## Discussion

Numerous evidence has shown the association between immune system or immune-related genes and patient prognosis in several solid tumors including breast cancer, non-small cell lung cancer, and ovarian cancer (15–17). In recent years, researchers have studied the survival benefits of immunotherapy in cervical cancer (2). In addition, some evidences also demonstrated the immune related signatures could predict the prognosis of cervical cancer (18). In this study, we established IPRS, a robust prognostic signature, based on 229 immune genes. Our results indicated that IPRS was significantly associated with cervical cancer patients’ OS in the TCGA cohort. The patients were stratified into high IPRS and low IPRS groups based on cut-off -74.30 of IPRS. High IPRS was associated with better survival, which suggests that clinicians should pay more attention to low IPRS. After adjusting for clinicopathological factors, IPRS remained an independent prognostic factor in the multivariable Cox model. In addition, we found that IPRS was also significantly correlated with PFS, and the prognosis of high IPRS group was also better than that in the low IPRS group. Further, we validated the predictive value of IPRS in the validation set and concluded that patients in the high IPRS group had better survival, which was similar to that of the training set. Further, the IPRS could further stratify patients in different clinically defined groups into subgroups with different survival outcome. These results indicated that IPRS is a robust prognostic signature.

Moreover, we performed a KEGG enrichment analysis for the 229 immune prognostic immune genes, which proved the association between cancer and clinical application potential. Cytokine-cytokine receptors are associated with inflammation, angiogenesis, and chemotaxis processes, and they inhibit tumor development and progression in addition to being effective in cancer treatment (3, 8). The natural killer cell-mediated cytotoxicity pathway is associated with NK cell activation, which controls tumor growth and kills tumors (19, 20). T cell receptor signaling pathway was related to the activation of T-cells in response to a cancer antigen (21). In addition, many studies showed that rheumatoid arthritis could increase the incidence of cervical cancer, and the prevalence of HPV infection was higher among women with autoimmune disease (22, 23). The incidence of cervical cancer was higher among women with Type I diabetes mellitus (24). MAPK signaling pathway and PI3K-Akt signaling pathway both lead to increased cancer cell invasiveness and facilitated cancer progression (25, 26).

Further, we found that IPRS and stage were independent prognostic factors. To improve the clinical application value of IPRS, a nomogram was constructed to predict mortality and instruct clinicians to adopt effective treatment measures.

Our study has several strengths. First, we developed an IPRS-based immune prognostic gene that proved to be an independent prognostic factor. Second, based on IPRS, patients could be stratified into high and low IPRS groups to benefit from different treatment. Third, we constructed a nomogram to predict mortality more accurately so that cervical cancer patients accept more immediate treatment. However, our study also has some limitations. First, the sample size used in the study is not large, which led to some related results such as cut-off values to change when reproducing results in other data. Second, we used public datasets for this study, and although IPRS was an independent prognostic factor, we could not validate its value in the actual dataset.

## Conclusion

We developed a robust prognostic signature IPRS, which could be used to predict patients’ survival outcome. Further studies are necessary to validate the prognostic value of IPRS in cervical cancer.

## Data Availability Statement

The datasets presented in this study can be found in online repositories. The names of the repository/repositories and accession number(s) can be found in the article/**Supplementary Material**.

## Author Contributions

YC and HL: conceptualization and methodology. Y-NP, HZ, YT and X-XC software and data curation and validation. YC and HL: writing—original draft. W-DZ and B-RX: writing—review and editing. All authors contributed to the article and approved the submitted version.

## Funding

This work was funded by the National Natural Science Foundation of China (No.81872430); Special Fund in China Postdoctoral Science Foundation (No.2019T120281), and Heilongjiang Province Postdoctoral Science Foundation (No.LBH-Z18109). Youth Scientific Research Fund project of the First Affiliated Hospital of USTC (Grant no.2020YJQN009).

## Conflict of Interest

The authors declare that the research was conducted in the absence of any commercial or financial relationships that could be construed as a potential conflict of interest.

## References

[1] Bray F Ferlay J Soerjomataram I Siegel RL Torre LA Jemal A. Global cancer statistics 2018: GLOBOCAN estimates of incidence and mortality worldwide for 36 cancers in 185 countries. CA: Cancer J Clin (2018) 68(6):394–424. 10.3322/caac.21492 30207593

[2] Ventriglia J Paciolla I Pisano C Cecere SC Di Napoli M Tambaro R. Immunotherapy in ovarian, endometrial and cervical cancer: state of the art and future perspectives. Cancer Treat Rev (2017) 59:109–16. 10.1016/j.ctrv.2017.07.008 28800469

[3] Shen S Wang G Zhang R Zhao Y Yu H Wei Y. Development and validation of an immune gene-set based Prognostic signature in ovarian cancer. EBioMedicine (2019) 40:318–26. 10.1016/j.ebiom.2018.12.054 PMC6412087 30594555

[4] Calon A Lonardo E Berenguer-Llergo A Espinet E Hernando-Momblona X Iglesias M. Stromal gene expression defines poor-prognosis subtypes in colorectal cancer. Nat Genet (2015) 47(4):320–9. 10.1038/ng.3225 25706628

[5] Li B Cui Y Diehn M Li R. Development and validation of an individualized immune prognostic signature in early-stage nonsquamous non–small cell lung cancer. JAMA Oncol (2017) 3(11):1529–37. 10.1001/jamaoncol.2017.1609 PMC5710196 28687838

[6] Ng SW Mitchell A Kennedy JA Chen WC McLeod J Ibrahimova N. A 17-gene stemness score for rapid determination of risk in acute leukaemia. Nature (2016) 540(7633):433–7. 10.1038/nature20598 27926740

[7] Szász AM Lánczky A Nagy Á Förster S Hark K Green JE. Cross-validation of survival associated biomarkers in gastric cancer using transcriptomic data of 1,065 patients. Oncotarget (2016) 7(31):49322. 10.18632/oncotarget.10337 27384994 PMC5226511

[8] Yang S Wu Y Deng Y Zhou L Yang P Zheng Y. Identification of a prognostic immune signature for cervical cancer to predict survival and response to immune checkpoint inhibitors. Oncoimmunology (2019) 8(12):e1659094. 10.1080/2162402X.2019.1659094 31741756 PMC6844304

[9] Huang H Liu Q Zhu L Zhang Y Lu X Wu Y. Prognostic value of preoperative systemic immune-inflammation index in patients with cervical cancer. Sci Rep (2019) 9(1):1–9. 10.1038/s41598-019-39150-0 30824727 PMC6397230

[10] Hastie T Tibshirani R Narasimhan B Chu G. impute: Imputation for microarray data. Bioinformatics (2001) 17(6):520–5. 10.1093/bioinformatics/17.6.520 11395428

[11] Bhattacharya S Andorf S Gomes L Dunn P Schaefer H Pontius J. ImmPort: disseminating data to the public for the future of immunology. Immunol Res (2014) 58(2-3):234–9. 10.1007/s12026-014-8516-1 24791905

[12] Sotiriou C Wirapati P Loi S Harris A Fox S Smeds J. Gene expression profiling in breast cancer: understanding the molecular basis of histologic grade to improve prognosis. J Natl Cancer Inst (2006) 98(4):262–72. 10.1093/jnci/djj052 16478745

[13] Zhang B Wu Q Li B Wang D Wang L Zhou YL. m 6 A regulator-mediated methylation modification patterns and tumor microenvironment infiltration characterization in gastric cancer. Mol Cancer (2020) 19(1):1–21. 10.1186/s12943-020-01170-0 32164750 PMC7066851

[14] Iasonos A Schrag D Raj GV Panageas KS. How to build and interpret a nomogram for cancer prognosis. J Clin Oncol (2008) 26(8):1364–70. 10.1200/JCO.2007.12.9791 18323559

[15] Mezquita L Auclin E Ferrara R Charrier M Remon J Planchard D. Association of the lung immune prognostic index with immune checkpoint inhibitor outcomes in patients with advanced non–small cell lung cancer. JAMA Oncol (2018) 4(3):351–7. 10.1001/jamaoncol.2017.4771 PMC5885829 29327044

[16] Schmidt M Böhm D Von Törne C Steiner E Puhl A Pilch H. The humoral immune system has a key prognostic impact in node-negative breast cancer. Cancer Res (2008) 68(13):5405–13. 10.1158/0008-5472.CAN-07-5206 18593943

[17] Zheng M Hu Y Gou R Liu O Nie X Li X. Identification of immune-enhanced molecular subtype associated with BRCA1 mutations, immune checkpoints and clinical outcome in ovarian carcinoma. J Cell Mol Med (2020) 24(5):2819–31. 10.1111/jcmm.14830 PMC7077593 31995855

[18] Wang J Li Z Gao A Wen Q Sun Y. The prognostic landscape of tumor-infiltrating immune cells in cervical cancer. Biomed Pharmacother (2019) 120:109444. 10.1016/j.biopha.2019.109444 31562978

[19] Paul S Lal G. The molecular mechanism of natural killer cells function and its importance in cancer immunotherapy. Front Immunol (2017) 8:1124. 10.3389/fimmu.2017.01124 28955340 PMC5601256

[20] Yoon SR Kim T-D Choi I. Understanding of molecular mechanisms in natural killer cell therapy. Exp Mol Med (2015) 47(2):e141–1. 10.1038/emm.2014.114 PMC4346487 25676064

[21] Huse M. The T-cell-receptor signaling network. J Cell Sci (2009) 122(9):1269–73. 10.1242/jcs.042762 19386893

[22] Simon TA Thompson A Gandhi KK Hochberg MC Suissa S. Incidence of malignancy in adult patients with rheumatoid arthritis: a meta-analysis. Arthritis Res Ther (2015) 17(1):212. 10.1186/s13075-015-0728-9 26271620 PMC4536786

[23] Kim SC Feldman S Moscicki A-B. Risk of human papillomavirus infection in women with rheumatic disease: cervical cancer screening and prevention. Rheumatology (2018) 57(suppl_5):v26–33. 10.1093/rheumatology/kex523 PMC6099129 30137592

[24] Zendehdel K Nyrén O Östenson C-G Adami H-O Ekbom A Ye W. Cancer incidence in patients with type 1 diabetes mellitus: a population-based cohort study in Sweden. J Natl Cancer Inst (2003) 95(23):1797–800. 10.1093/jnci/djg105 14652242

[25] Shukla S MacLennan GT Hartman DJ Fu P Resnick MI Gupta S. Activation of PI3K-Akt signaling pathway promotes prostate cancer cell invasion. Int J Cancer (2007) 121(7):1424–32. 10.1002/ijc.22862 17551921

[26] Sumimoto H Imabayashi F Iwata T Kawakami Y. The BRAF–MAPK signaling pathway is essential for cancer-immune evasion in human melanoma cells. J Exp Med (2006) 203(7):1651–6. 10.1084/jem.20051848 PMC2118331 16801397

