# Supplementary material for: Development and Validation of a Prognostic Signature Based on Immune Genes in Cervical Cancer
Source: Front Oncol. 2021 Mar 17;11:616530. doi: 10.3389/fonc.2021.616530 (PMC8029986; doi:10.3389/fonc.2021.616530)
Supplement: Supplementary file 1 [file DataSheet_1.docx]

Table S1 The immune genes associated with prognosis in cervical cancer

| Protective genes | | | | | |  | Dangerous genes | | | | |  |
| --- | --- | --- | --- | --- | --- | --- | --- | --- | --- | --- | --- | --- |
| num | gene | num | gene | num | gene |  | num | | gene | num | gene |  |
| 1 | ANGPTL6 | 49 | CXCL14 | 97 | KLRC2 |  | 1 | | ADIPOQ | 49 | NOD1 |  |
| 2 | APOBEC3C | 50 | CXCR3 | 98 | KLRD1 |  | 2 | | ANGPTL4 | 50 | NRP1 | |
| 3 | APOBEC3F | 51 | CXCR6 | 99 | KLRK1 |  | 3 | | ANGPTL5 | 51 | OPRD1 |  |
| 4 | APOBEC3G | 52 | CYSLTR1 | 100 | LAT |  | 4 | | APLN | 52 | OSM |  |
| 5 | APOBEC3H | 53 | CYSLTR2 | 101 | LCK |  | 5 | | BMP10 | 53 | PAEP |  |
| 6 | APOD | 54 | DES | 102 | LCN6 |  | 6 | | CANX | 54 | PDF |  |
| 7 | BLNK | 55 | DHX58 | 103 | LTA |  | 7 | | CCL20 | 55 | PDK1 |  |
| 8 | BMP7 | 56 | DUOX1 | 104 | LYZ |  | 8 | | CRP | 56 | PGC |  |
| 9 | BTK | 57 | FAM3B | 105 | MAP3K14 | | 9 | | CSF2 | 57 | PLAU |  |
| 10 | C5AR1 | 58 | FASLG | 106 | NCR1 |  | 10 | | CXCL1 | 58 | PPARG |  |
| 11 | C8G | 59 | FGFR2 | 107 | NFATC1 |  | 11 | | CXCL2 | 59 | PROK2 |  |
| 12 | CAMP | 60 | FLT3LG | 108 | NFATC2 |  | 12 | | CXCL3 | 60 | PTGS2 |  |
| 13 | CCL17 | 61 | GHRL | 109 | NFYB |  | 13 | | CXCL5 | 61 | PTX3 |  |
| 14 | CCL19 | 62 | GMFG | 110 | NGFR |  | 14 | | DCD | 62 | R3HDML |  |
| 15 | CCL22 | 63 | GRAP2 | 111 | PDCD1 |  | 15 | | DEFA3 | 63 | REG1A |  |
| 16 | CCL5 | 64 | GZMB | 112 | PIK3CD |  | 16 | | DEFB133 | 64 | REG3G |  |
| 17 | CCR5 | 65 | HCK | 113 | PIK3R2 |  | 17 | | DLL4 | 65 | RETN |  |
| 18 | CCR7 | 66 | HCST | 114 | PIK3R3 |  | 18 | | DMBT1 | 66 | RETNLB |  |
| 19 | CD19 | 67 | HLA-DMB | 115 | PIK3R5 |  | 19 | EGFR | | 67 | S100A10 |  |
| 20 | CD1C | 68 | HLA-DPA1 | 116 | PPP3CC |  | 20 | EREG | | 68 | SBDS |  |
| 21 | CD1E | 69 | HLA-DPB1 | 117 | PRDX2 |  | 21 | ESM1 | | 69 | SEMA3A |  |
| 22 | CD209 | 70 | HLA-DQA1 | 118 | PRF1 |  | 22 | FABP4 | | 70 | SEMA3C |  |
| 23 | CD244 | 71 | HLA-DQB1 | 119 | PRKCB |  | 23 | FGF5 | | 71 | SEMA4G |  |
| 24 | CD247 | 72 | HLA-DRA | 120 | PSMC3 |  | 24 | FGF9 | | 72 | SERPINA3 |  |
| 25 | CD28 | 73 | HLA-DRB1 | 121 | PTGDS |  | 25 | FURIN | | 73 | SHC1 |  |
| 26 | CD3D | 74 | ICOS | 122 | PTGER4 |  | 26 | GALP | | 74 | SPP1 |  |
| 27 | CD3E | 75 | IFI30 | 123 | PTK2B |  | 27 | GHSR | | 75 | STC1 |  |
| 28 | CD3G | 76 | IFNG | 124 | PTPN6 |  | 28 | GUCA2A | | 76 | TFRC |  |
| 29 | CD4 | 77 | IL10RA | 125 | PTPRC |  | 29 | HSP90AB1 | | 77 | TGFA |  |
| 30 | CD48 | 78 | IL12A | 126 | RABEP2 |  | 30 | HSPA5 | | 78 | TINAGL1 |  |
| 31 | CD72 | 79 | IL12RB1 | 127 | RFXANK |  | 31 | HSPA8 | | 79 | TNF |  |
| 32 | CD74 | 80 | IL16 | 128 | SEMA4D |  | 32 | IFNA17 | | 80 | TNFRSF10D |  |
| 33 | CD79A | 81 | IL17RB | 129 | SH2D1A |  | 33 | IFNA2 | | 81 | TNFRSF11B |  |
| 34 | CD79B | 82 | IL18R1 | 130 | SOCS1 |  | 34 | IGF2R | | 82 | TNFRSF12A |  |
| 35 | CD86 | 83 | IL18RAP | 131 | TLR1 |  | 35 | IL17C | | 83 | TUBB3 |  |
| 36 | CD8A | 84 | IL21R | 132 | TMSB15A | | 36 | IL1A | | 84 | UCN3 |  |
| 37 | CD8B | 85 | IL22RA2 | 133 | TNFRSF13B | | 37 | IL1B | | 85 | VEGFA |  |
| 38 | CETP | 86 | IL27 | 134 | TNFRSF13C | | 38 | ILK | | 86 | ZC3HAV1L |  |
| 39 | CHIT1 | 87 | IL2RG | 135 | TNFRSF1B | | 39 | INS | |  |  |  |
| 40 | CKLF | 88 | IL34 | 136 | TNFRSF9 |  | 40 | JUN | |  |  |  |
| 41 | CMKLR1 | 89 | IL3RA | 137 | TNFSF13B | | 41 | LBP | |  |  |  |
| 42 | CORT | 90 | IL9R | 138 | TSHR |  | 42 | LCN9 | |  |  |  |
| 43 | CRLF2 | 91 | INSL3 | 139 | TYK2 |  | 43 | LEPR | |  |  |  |
| 44 | CSF2RA | 92 | ISG20 | 140 | TYROBP |  | 44 | LIF | |  |  |  |
| 45 | CSF2RB | 93 | ITGAL | 141 | VCAM1 |  | 45 | LMBR1L | |  |  |  |
| 46 | CSK | 94 | ITGB2 | 142 | XCR1 |  | 46 | MICA | |  |  |  |
| 47 | CTLA4 | 95 | ITK | 143 | ZAP70 |  | 47 | NAMPT | |  |  |  |
| 48 | CX3CL1 | 96 | JUND |  |  |  | 48 | NDRG1 | |  |  |  |

Table S2 Pathway enrichment analysis for IPRS genes from KEGG

| ID | Pathway name | GeneRatio | Raw *P* value | Adjusted *P* value |
| --- | --- | --- | --- | --- |
| hsa04060 | Cytokine-cytokine receptor interaction | 57/193 | 2.92E-37 | 6.04E-35 |
| hsa04650 | Natural killer cell mediated cytotoxicity | 34/193 | 1.62E-26 | 1.68E-24 |
| hsa04660 | T cell receptor signaling pathway | 29/193 | 1.14E-23 | 7.88E-22 |
| hsa05323 | Rheumatoid arthritis | 26/193 | 2.51E-21 | 1.30E-19 |
| hsa04940 | Type I diabetes mellitus | 19/193 | 3.09E-20 | 1.15E-18 |
| hsa04658 | Th1 and Th2 cell differentiation | 25/193 | 3.34E-20 | 1.15E-18 |
| hsa04659 | Th17 cell differentiation | 26/193 | 1.26E-19 | 3.73E-18 |
| hsa04640 | Hematopoietic cell lineage | 25/193 | 2.40E-19 | 6.20E-18 |
| hsa05340 | Primary immunodeficiency | 17/193 | 2.49E-18 | 5.72E-17 |
| hsa04612 | Antigen processing and presentation | 22/193 | 2.82E-18 | 5.84E-17 |
| hsa04061 | Viral protein interaction with cytokine and cytokine receptor | 24/193 | 4.78E-18 | 8.99E-17 |
| hsa05332 | Graft-versus-host disease | 17/193 | 2.03E-17 | 3.49E-16 |
| hsa05321 | Inflammatory bowel disease | 19/193 | 3.01E-16 | 4.80E-15 |
| hsa05330 | Allograft rejection | 15/193 | 2.64E-15 | 3.90E-14 |
| hsa05166 | Human T-cell leukemia virus 1 infection | 30/193 | 3.90E-15 | 5.38E-14 |
| hsa05235 | PD-L1 expression and PD-1 checkpoint pathway in cancer | 20/193 | 1.30E-14 | 1.69E-13 |
| hsa05320 | Autoimmune thyroid disease | 16/193 | 4.18E-14 | 5.09E-13 |
| hsa04630 | JAK-STAT signaling pathway | 25/193 | 5.74E-14 | 6.60E-13 |
| hsa04668 | TNF signaling pathway | 21/193 | 1.29E-13 | 1.40E-12 |
| hsa04064 | NF-kappa B signaling pathway | 20/193 | 3.08E-13 | 3.18E-12 |
| hsa05140 | Leishmaniasis | 17/193 | 1.88E-12 | 1.85E-11 |
| hsa04380 | Osteoclast differentiation | 21/193 | 1.99E-12 | 1.87E-11 |
| hsa04062 | Chemokine signaling pathway | 25/193 | 2.98E-12 | 2.69E-11 |
| hsa04672 | Intestinal immune network for IgA production | 14/193 | 4.03E-12 | 3.48E-11 |
| hsa05152 | Tuberculosis | 24/193 | 5.01E-12 | 4.15E-11 |
| hsa05169 | Epstein-Barr virus infection | 25/193 | 9.40E-12 | 7.49E-11 |
| hsa05170 | Human immunodeficiency virus 1 infection | 24/193 | 1.69E-10 | 1.30E-09 |
| hsa04657 | IL-17 signaling pathway | 16/193 | 5.47E-10 | 4.01E-09 |
| hsa05164 | Influenza A | 21/193 | 5.61E-10 | 4.01E-09 |
| hsa04662 | B cell receptor signaling pathway | 15/193 | 6.76E-10 | 4.67E-09 |
| hsa05146 | Amoebiasis | 16/193 | 1.91E-09 | 1.28E-08 |
| hsa04514 | Cell adhesion molecules | 19/193 | 2.06E-09 | 1.33E-08 |
| hsa05162 | Measles | 18/193 | 4.35E-09 | 2.73E-08 |
| hsa05167 | Kaposi sarcoma-associated herpesvirus infection | 21/193 | 5.25E-09 | 3.19E-08 |
| hsa05145 | Toxoplasmosis | 16/193 | 7.78E-09 | 4.60E-08 |
| hsa05416 | Viral myocarditis | 12/193 | 1.27E-08 | 7.30E-08 |
| hsa04625 | C-type lectin receptor signaling pathway | 15/193 | 2.04E-08 | 1.14E-07 |
| hsa05163 | Human cytomegalovirus infection | 21/193 | 7.96E-08 | 4.34E-07 |
| hsa05142 | Chagas disease | 14/193 | 1.17E-07 | 6.20E-07 |
| hsa04620 | Toll-like receptor signaling pathway | 14/193 | 1.50E-07 | 7.75E-07 |
| hsa05135 | Yersinia infection | 15/193 | 4.19E-07 | 2.12E-06 |
| hsa05310 | Asthma | 8/193 | 4.54E-07 | 2.24E-06 |
| hsa05150 | Staphylococcus aureus infection | 12/193 | 2.67E-06 | 1.29E-05 |
| hsa04010 | MAPK signaling pathway | 21/193 | 6.52E-06 | 3.07E-05 |
| hsa04151 | PI3K-Akt signaling pathway | 23/193 | 1.12E-05 | 5.14E-05 |
| hsa05144 | Malaria | 8/193 | 2.10E-05 | 9.43E-05 |
| hsa05143 | African trypanosomiasis | 7/193 | 2.24E-05 | 9.88E-05 |
| hsa04933 | AGE-RAGE signaling pathway in diabetic complications | 11/193 | 2.43E-05 | 1.05E-04 |
| hsa04621 | NOD-like receptor signaling pathway | 15/193 | 2.63E-05 | 1.11E-04 |
| hsa04014 | Ras signaling pathway | 17/193 | 3.74E-05 | 1.55E-04 |
| hsa05120 | Epithelial cell signaling in Helicobacter pylori infection | 9/193 | 3.91E-05 | 1.59E-04 |
| hsa05134 | Legionellosis | 8/193 | 5.59E-05 | 2.22E-04 |
| hsa04370 | VEGF signaling pathway | 8/193 | 7.20E-05 | 2.81E-04 |
| hsa05322 | Systemic lupus erythematosus | 12/193 | 9.41E-05 | 3.61E-04 |
| hsa04930 | Type II diabetes mellitus | 7/193 | 9.72E-05 | 3.66E-04 |
| hsa01521 | EGFR tyrosine kinase inhibitor resistance | 9/193 | 1.03E-04 | 3.80E-04 |
| hsa05161 | Hepatitis B | 13/193 | 1.27E-04 | 4.63E-04 |
| hsa04012 | ErbB signaling pathway | 9/193 | 1.82E-04 | 6.48E-04 |
| hsa04066 | HIF-1 signaling pathway | 10/193 | 2.64E-04 | 9.27E-04 |
| hsa04145 | Phagosome | 12/193 | 2.70E-04 | 9.33E-04 |
| hsa04923 | Regulation of lipolysis in adipocytes | 7/193 | 3.44E-04 | 1.17E-03 |
| hsa04360 | Axon guidance | 13/193 | 3.82E-04 | 1.28E-03 |
| hsa04210 | Apoptosis | 11/193 | 3.95E-04 | 1.30E-03 |
| hsa05133 | Pertussis | 8/193 | 4.33E-04 | 1.40E-03 |
| hsa05418 | Fluid shear stress and atherosclerosis | 11/193 | 4.76E-04 | 1.52E-03 |
| hsa04015 | Rap1 signaling pathway | 14/193 | 4.89E-04 | 1.52E-03 |
| hsa05215 | Prostate cancer | 9/193 | 4.93E-04 | 1.52E-03 |
| hsa04932 | Non-alcoholic fatty liver disease | 11/193 | 9.04E-04 | 2.75E-03 |
| hsa04664 | Fc epsilon RI signaling pathway | 7/193 | 1.13E-03 | 3.39E-03 |
| hsa04670 | Leukocyte transendothelial migration | 9/193 | 1.58E-03 | 4.67E-03 |
| hsa04960 | Aldosterone-regulated sodium reabsorption | 5/193 | 1.74E-03 | 5.06E-03 |
| hsa05214 | Glioma | 7/193 | 2.01E-03 | 5.79E-03 |
| hsa05160 | Hepatitis C | 10/193 | 4.32E-03 | 1.22E-02 |
| hsa05210 | Colorectal cancer | 7/193 | 4.37E-03 | 1.22E-02 |
| hsa05223 | Non-small cell lung cancer | 6/193 | 5.53E-03 | 1.53E-02 |
| hsa04915 | Estrogen signaling pathway | 9/193 | 5.72E-03 | 1.56E-02 |
| hsa05211 | Renal cell carcinoma | 6/193 | 5.94E-03 | 1.60E-02 |
| hsa04917 | Prolactin signaling pathway | 6/193 | 6.37E-03 | 1.67E-02 |
| hsa05230 | Central carbon metabolism in cancer | 6/193 | 6.37E-03 | 1.67E-02 |
| hsa04666 | Fc gamma R-mediated phagocytosis | 7/193 | 6.71E-03 | 1.74E-02 |
| hsa05218 | Melanoma | 6/193 | 7.30E-03 | 1.86E-02 |
| hsa04935 | Growth hormone synthesis, secretion and action | 8/193 | 7.55E-03 | 1.90E-02 |
| hsa05168 | Herpes simplex virus 1 infection | 21/193 | 7.62E-03 | 1.90E-02 |
| hsa05224 | Breast cancer | 9/193 | 8.56E-03 | 2.11E-02 |
| hsa01522 | Endocrine resistance | 7/193 | 8.87E-03 | 2.16E-02 |
| hsa05212 | Pancreatic cancer | 6/193 | 9.44E-03 | 2.27E-02 |
| hsa04926 | Relaxin signaling pathway | 8/193 | 1.20E-02 | 2.85E-02 |
| hsa05213 | Endometrial cancer | 5/193 | 1.22E-02 | 2.87E-02 |
| hsa05020 | Prion disease | 13/193 | 1.37E-02 | 3.19E-02 |
| hsa04217 | Necroptosis | 9/193 | 1.39E-02 | 3.19E-02 |
| hsa04213 | Longevity regulating pathway - multiple species | 5/193 | 1.60E-02 | 3.63E-02 |
| hsa04929 | GnRH secretion | 5/193 | 1.81E-02 | 4.07E-02 |
| hsa04211 | Longevity regulating pathway | 6/193 | 1.95E-02 | 4.34E-02 |
| hsa04510 | Focal adhesion | 10/193 | 2.22E-02 | 4.89E-02 |

Table S3 Cox regression analysis for the IPRS and clinical characteristics in TCGA

| variables | HR (95% CI) | *pvalue* |
| --- | --- | --- |
| IPRS |  |  |
| High | reference |  |
| Low | 4.07(2.29 to 7.23) | <0.001 |
| Stage |  |  |
| I | reference |  |
| II | 0.72(0.33 to 1.57) | 0.411 |
| III | 1.01(0.46 to 2.22) | 0.987 |
| IV | 3.02(1.37 to 6.62) | 0.006 |
| Grade |  |  |
| I/II | reference |  |
| III/IV | 1.1(0.63 to 1.93) | 0.738 |
| Age | 1.01(0.99 to 1.03) | 0.296 |

Table S4 Cox regression analysis for the IPSOV and clinical characteristics in GEO

| variables | HR (95% CI) | *pvalue* |
| --- | --- | --- |
| IPRS |  |  |
| High | reference |  |
| Low | 2.67(1.11 to 6.39) | 0.028 |
| Stage |  |  |
| I | reference |  |
| II | 1.65(0.76 to 3.62) | 0.208 |


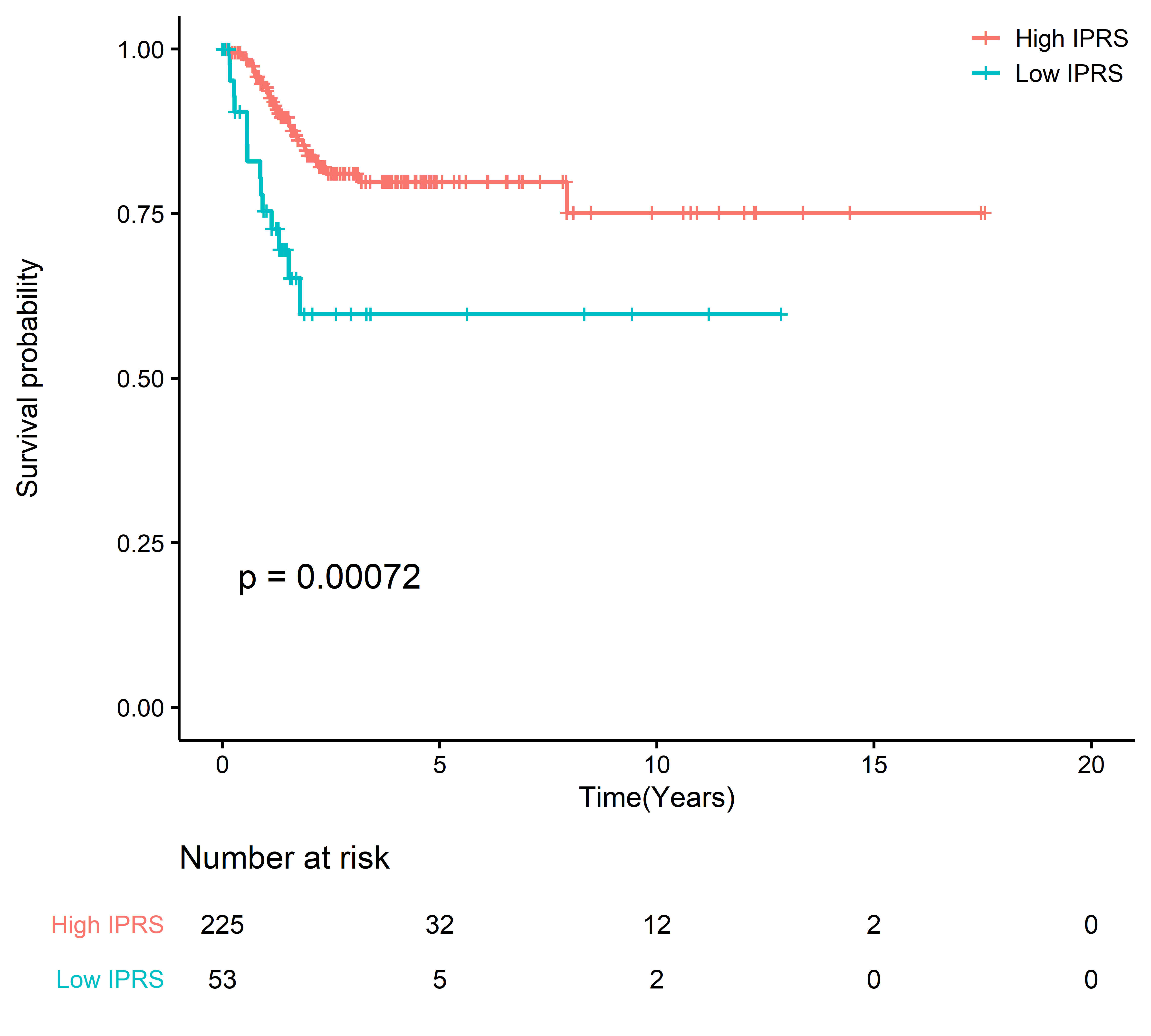


**Figure S1 Survival curve of PFS between high IRPS and low IRPS**
